# Changes in Protein Expression in Two Cholangiocarcinoma Cell Lines Undergoing Formation of Multicellular Tumor Spheroids *In Vitro*

**DOI:** 10.1371/journal.pone.0118906

**Published:** 2015-03-10

**Authors:** Carlo Mischiati, Blendi Ura, Leda Roncoroni, Luca Elli, Carlo Cervellati, Monica Squerzanti, Dario Conte, Luisa Doneda, Patrizia Polverino de Laureto, Giorgia de Franceschi, Roberta Calza, Carlos A. Barrero, Salim Merali, Carlo Ferrari, Carlo M. Bergamini, Enzo Agostinelli

**Affiliations:** 1 Department of Biomedical and Surgical Sciences, University of Ferrara, Ferrara, Italy; 2 Institute for Maternal and Child Health, IRCCS Burlo Garofalo, University of Trieste, Trieste, Italy; 3 Center for Prevention and Diagnosis of Coeliac Disease/Gastroenterology 2, Fondazione IRCCS Cà Granda, Ospedale Maggiore Policlinico, Milano, Italy; 4 Department of Biomedical, Surgical and Odontoiatric Sciences, University of Milano, Milano, Italy; 5 CRIBI Centre of Innovative Biotechnologies, University of Padua, Padua, Italy; 6 Moulder Center for Drug Discovery Research, Temple University, School of Pharmacy, Philadelphia, Pennsylvania, United States of America; 7 Department of Clinical and Molecular Sciences, Faculty of Medicine, Le Marche Polytechnic University, Ancona, Italy; 8 Istituto Pasteur, Fondazione Cenci Bolognetti and Department of Biochemical Sciences “A. Rossi Fanelli”, La Sapienza University of Rome and CNR, Biology and Molecular Pathology Institutes, Rome, Italy; National Cancer Institute, NIH, UNITED STATES

## Abstract

Epithelial-to-Mesenchymal Transition (EMT) is relevant in malignant growth and frequently correlates with worsening disease progression due to its implications in metastases and resistance to therapeutic interventions. Although EMT is known to occur in several types of solid tumors, the information concerning tumors arising from the epithelia of the bile tract is still limited. In order to approach the problem of EMT in cholangiocarcinoma, we decided to investigate the changes in protein expression occurring in two cell lines under conditions leading to growth as adherent monolayers or to formation of multicellular tumor spheroids (MCTS), which are considered culture models that better mimic the growth characteristics of *in-vivo* solid tumors. In our system, changes in phenotypes occur with only a decrease in transmembrane E-cadherin and vimentin expression, minor changes in the transglutaminase protein/activity but with significant differences in the proteome profiles, with declining and increasing expression in 6 and in 16 proteins identified by mass spectrometry. The arising protein patterns were analyzed based on canonical pathways and network analysis. These results suggest that significant metabolic rearrangements occur during the conversion of cholangiocarcinomas cells to the MCTS phenotype, which most likely affect the carbohydrate metabolism, protein folding, cytoskeletal activity, and tissue sensitivity to oxygen.

## Introduction

In the late 1980s researchers discovered that epithelial cells may differentiate to assume fibroblast-like appearance and behavior [1]. This process is termed Epithelial to Mesenchymal Transition (EMT) and occurs under physiological and pathological conditions (developmental growth, tissue repair and cancer invasion) with the reprogramming of protein expression in relation to cell differentiation [2, 3]. Epithelial cells thus convert into fibroblast-like mesenchymal cells that gain motility from cytoskeletal rearrangement, the disruption of intercellular contacts, the down-regulation of cell adhesive molecules and the increased activity of proteinases to favor the movement of the cells to new locations, where they may reverse the EMT process. Cytokines, primarily TGFβ, control the occurrence of EMT by involving bone marrow-derived cells, although local and mechanical stimuli may also be relevant [4].

Three forms of EMT have been described [2]: type-1 is involved in embryonic growth, type-2 is involved in wound repair and type-3 is involved in tumor growth. In type-3 EMT, undifferentiated mesenchymal-like cancer cells gain easier access to blood vessels for tumor spreading. This mechanism can account for major differences in the biological and clinical behavior of primary *vs*. secondary tumor growth, which are respectively promoted in epithelial and fibroblast-like cancer cells, as proven in the Head-Neck Squamous Cell Carcinoma (HNSCC) [5]. Thus, the typical morphologic heterogeneity of aggressive tumors due to cellular de-differentiation depends either on EMT or on the intrinsic properties of tumors that contain well and poorly differentiated cells. The latter should be considered cancer stem cells that are definitely more capable of invading and metastasizing. In this context, differences in the regulation of protein expression in primary and metastatic lesions are easily understandable. Some issues, such as the spread of ovarian tumors within the peritoneal cavity as ascites tumors and the acquisition of resistance to classic chemo- or radiotherapy, are associated with EMT [6–8]. EMT has been described in several types of solid tumors, including HNSCC, prostate, pancreatic and lung cancer ([9, 10] and references therein).

Multicellular Tumor Spheroids (MCTS), which are generated by the *in-vitro* conversion of adherent cells growing in 2D into spheroid cell aggregates [11, 12], are used as a model in studies of tumor biology since they better mimic the growth characteristics of *in-vivo* solid tumors, locally characterized by hypoxia, acidosis, and nutrient deprivation, which collectively lead to tumor genetic and adaptive changes [13]. Although the changes in the patterns of cellular aggregation can be triggered also by adding up cytokines to culture media, even the simple hypoxic and mechanical stimulations promote MCTS formation in HepG2 hepatocellular carcinoma [14] and breast cancer MCF-7 cultured cells [15] undergoing EMT as proved by the upregulation of vimentin and loss of E-cadherin expression [13].

The issues of tumor growth kinetics and EMT have been the subject of limited studies on tumors of the biliary tract [16], despite the severity of Cholangiocarcinomas (CC). CCs are highly invasive tumors that originate from the epithelial cell lining of the bile ducts and account for 3% of all gastrointestinal tumors [17]. Primary lesions affect the gallbladder, the hepatic and the common duct, the intraduodenal portion of the common duct or even the intrahepatic bile ducts. CCs are not easily diagnosed and are often discovered at an advanced stage because symptoms are rather unspecific (abdominal pain, jaundice, digestive disturbances, itching, laboratory signs of cholestasis) and arise when the obstruction of the biliary system is established, making surgery suitable in only a limited number of patients, even though the critical location usually permits the detection of tumors that are still small in size. Chemo- and radiotherapy of CCs have proved disappointing in terms of survival in inoperable patients, and the development of new therapies is also urgently needed for patients with pathologies at high risk to develop CCs, as in the case of primary sclerosing cholangitis.

In this perspective we selected as an experimental system the CC cell lines SK-ChA-1 and MZ-ChA-1, to investigate these topics. The above cell lines form MCTS during culture under stirring as opposed to resting [18,19], displaying features that remind EMT, although the correlation between changes in cell aggregation and the process of EMT is still a controversial issue at least *in-vivo* [20]. The approach we followed was the quantitative assessment by proteomic analysis of the perturbation of protein expression in these CC cell lines, growing as 3D compared with 2D cell cultures.

## Materials and Methods

### Cell culture

The CC cell lines SK-ChA-1 and MZ-ChA-1 were originally isolated by Prof. Alexander Knuth from undifferentiated and well-differentiated primary CC of the extrahepatic bile ducts, respectively [18]. Samples of these cell lines were obtained from the Cancer Immunology Laboratory of the Department of Oncology at the University Hospital of Zürich. They were cultured as previously described [19] under different experimental settings to obtain either two-dimensional (2D) or three-dimensional (3D) cultures (which are also called multicellular tumor spheroids, MCTS). In the 2D setting, both cell lines were grown in IMDM (Iskove modified Dulbecco’s medium, Gibco) supplemented with 1 U/ml penicillin, 1 mg/ml streptomycin, 4 mM L-glutamine and 10% fetal bovine serum in a humidified 5% CO_2_ incubator at 37°C in T75 flasks. They were collected after 4 days in culture. In the 3D culture approach, growth was started by seeding 2x10^5^ cells/ml in 15 ml of complete medium in polycarbonate Erlenmeyer flasks (Corning) incubated in a gyratory rotation incubator (60 rev/min) at 37°C in an air atmosphere. Homotypical aggregations became visible after 4 days of culture, and MCTS’s were usually complete within 7 days (mean diameter ± standard deviation, 270 ± 53.5 μm), although a longer time was required in some occasions.

### Cell lysis and two-dimensional (2D)-PAGE analysis of proteins

The cells obtained from the 2D and 3D cultures were collected by centrifugation and lysed by treatment with TUC buffer (2 M thiourea, 7 M Urea, 4% CHAPS, 40 mM Tris, pH 9.0) supplemented with a cocktail of protease inhibitors. One-hundred thirty microliters of lysis buffer were used for 1 million cells. After determining the protein content using the Bradford assay [21], 300 μg protein was loaded onto a IEF strip with a non-linear range between pH 3 and 10 (3–10 NL IPG strip of 7 cm in length, Biorad) that was focused for 12 hours until reaching the maximum voltage of 4000V to obtain a total of 60000 Volt h. After reducing the thiol groups using 1% DTT and alkylating them with 4% iodoacetamide, the second dimension was run on slab polyacrylamide gels (12.5%) at 200V until the bromophenol marker reached the bottom of the gel to avoid the loss of small MW peptides. In the case of the 3D spheroids, lysis was performed with 150 μl the same lysis buffer, and the protein load on the IEF strips was based only on the protein assay. After fixing, the polyacrylamide gels were washed twice with bi-distillated water and stained with colloidal Coomassie, scanned with Molecular Imager Pharos FX Systems and analyzed using the ProteomeWeaver 4 program (Biorad). After normalizing the spot intensity with the same program, proteins with significant changes in expression were further processed for mass spectroscopy analysis (MS). All studies were performed in triplicate.

### Identification of target proteins by mass spectrometry and bioinformatic analysis

Spots from differentially expressed proteins were excised and subjected to trypsin digestion for MS-based analysis as previously described [22]. Briefly, the gel fragments were rinsed in buffered acetonitrile and dried. Following additional thiol group reduction and alkylation, the proteins were digested overnight with 12.5 ng/μl trypsin, solubilized in aqueous formic acid, resolved by LC-MS/MS and examined on a 6520 Q-TOF mass spectrometer (Agilent Technologies, Santa Clara, CA, USA) coupled to a chip-based chromatographic interface. A Large Capacity Chip (C18, 150 μm × 75 μm) with an enrichment column (C18, 9 mm, 160 nl volume) was used to separate peptides at a flow rate of 0.3 μl/min. Water/formic acid 0.1% and acetonitrile/formic acid 0.1% were used as eluents A and B, respectively. The chromatographic separation was achieved with a gradient of B from 5% to 50% in 20 mins. The raw data files were converted into Mascot Generic Format (MGF) with the MassHunter Qualitative Analysis Software version B.03.01 (Agilent Technologies) and analyzed using the Mascot Search Engine version 2.2.4 (Matrix Science). The pattern of proteolytic cleavage by trypsin was adjusted at the basic amino acids lysine and arginine, which are specifically recognized by protease, assuming the possibility of 1 missed cleavage per peptide chain and using a mass tolerance window of 1.2 Da for peptides and 0.6 Da for fragment ion matches. The carbamido-methylation of cysteine was set as fixed modification, and methionine oxidation was set as variable modification. Proteins were considered positive hits if at least 2 peptides per protein were identified with high confidence (*p* <0.05) by the automatic procedure of the Mascot Search engine (see above). Proteins displaying higher than 2-fold changes in expression were selected for a standard biological functions cluster and network analysis using the Ingenuity Pathways Analysis (IPA) software as previously described [23]. To build up the network, the Ingenuity knowledge database (http://www.ingenuity.com/index/html) was queried for physical and functional interactions between the submitted proteins and all other proteins in the database. IPA was optimized to include up to 35 proteins in a network.

### Western blot analysis

Cells grown as monolayers or as MCTS were lysed in TUC buffer as described above, and the extracts were submitted to SDS-PAGE on a 10% slab gel via the Laemmli procedure [24]. Four independent experiments were performed and analyzed via Western blotting. Briefly, the separated proteins were transferred to a nitrocellulose membrane in a blotting chamber, and the residual binding sites on the membrane were blocked by treatment with defatted dry milk proteins before staining with specific monoclonal or polyclonal primary antibodies and peroxidase-conjugated secondary antibodies and development with the luminol substrate. Primary antibodies against transglutaminase type 2 (Tgase2, Covalab, Lyon, France), cadherin E (Pierce, Rockford, Illinois), vimentin (UBI, Lake Place, NY, USA), and actin (Santa Cruz, Dallas, TX, USA) were employed.

### Assay of activity of Transglutaminase and other enzymes of the carbohydrate metabolism

For these experiments, the cells were lysed with two volumes (v/w) of 50 mM Tris, 0.5 mM EDTA, 1 mM PMSF and 1 mM mercaptoethanol at pH 7.5 via 3 cycles of freezing/thawing followed by vortex stirring. After centrifugation in a refrigerated Eppendorf centrifuge at 12,000 rpm for 15 minutes, the supernatants were saved to estimate the protein concentration [21] and enzyme activity after supplementing with 0.5 mM DTT to fully activate transglutaminase. The activity was measured with a filter paper assay that employed radioactive putrescine and dimethylcasein as the amine and protein acceptor substrates, as in ref. [25], at saturating (5 mM) and at sub-saturating (0.5 mM) concentrations of free calcium. In this last instance, we also included parallel assays in the presence of 0.2 mM Ca.GTP in order to assess the differential sensitivity to the effects of ligands [26], as discussed further on. In additional experiments, we measured the activities of other enzymes of the carbohydrate metabolism (pyruvate kinase, aldolase, malate dehydrogenase and isocitrate dehydrogenase) via established procedures [27–30].

### Statistical analysis

The statistical analysis was performed using a paired Student’s t-test comparing proteins spots from cells obtained from 2D and MCTS culture replicates to identify those spots among the selected samples that were differently modulated in the MCTS. The level of significance was set at p <0.05 for the proteins presented in Table 1. IPA uses a z-score algorithm to make predictions. The z-score algorithm is designed to reduce the chance that random data will generate significant predictions. Details regarding the Statistics for IPA analysis can be found at: http://www.ingenuity.com/index/html.

**Table 1:** Differential expression of proteins identified by 2D PAGE.

*Increasing Proteins*				
*Protein ID*	*Entry Name*	*Protein Name*	*Cell Line*	*Relative Intensity*
P14625	ENPL_HUMAN	Endoplasmin precursor	SK/MZ	2.4
P02768	ALBU_HUMAN	Serum albumin precursor	SK	2.0
P07237	PDIA1_HUMAN	Protein disulfide-isomerase precursor	SK	5.0
P36952	SPB5_HUMAN	Maspin	SK	3.1
P60174	TPIS_HUMAN	Triosephosphate isomerase	SK	3.0
P08294	SODE_HUMAN	Superoxide dismutase 3	SK	3.1
O75874	IDHC_HUMAN	IDH1(isocitrate dehydrogenase)	SK	3.0
Q9Y4L1	HYOU1_HUMAN	150 kDa oxygen-regulated protein	MZ	2.1
P08107	HSP71_HUMAN	Hsp 70	MZ	2.0
P07237	PDIA1_HUMAN	Protein disulfide-isomerase precursor	MZ	2.0
Q53G71	Q53G71_HUMAN	Calreticulin variant	MZ	2.0
P10809	CH60_HUMAN	Chaperonin (Hsp 60)	SK	2.0
O75874	IDHC_HUMAN	Isocitrate dehydrogenase	SK/MZ	2.0
Q8IY26	PPAC2_HUMAN	Phosphatidic acid phosphatase type 2	SK	2.0
P27797	CALR_HUMAN	Calreticulin	MZ	5.0
P04406	G3P_HUMAN	Glyceraldehyde phosphate dehydrogenase	MZ	3.0

*Decreasing Proteins*				
*Protein ID*	*Entry Name*	*Protein name*	*Cell Line*	*Relative Intensity*
P15311	EZRI_HUMAN	Villin 2 (Ezrin)	SK	0.1
P14618	KPYM_HUMAN	Pyruvate kinase isozymes M1/M2	SK	0.4
P08670	VIME_HUMAN	Vimentin	SK/MZ	0.1
Q04695	K1C17_HUMAN	Keratin type 1- Cytoskeletal 17	MZ	0.2
P08727	K1C19_HUMAN	Keratin type 1- Cytoskeletal 19	MZ	0.3
P46108	CRK_HUMAN	CRK II (proto-oncogene c-CRK)	MZ	0.1

Following bi-dimensional separation of SKCHA1(SK) and MZCHA1(MZ) cell lysates, the amount of protein present in selected spots of adherent and spheroid cells was determined by densitometry and values expressed as spheroid/adherent ratio (with statistically significant p > 0.05). A ratio >2 indicates that the protein is upregulated during EMT while a ratio <0.5 denotes proteins downregulated during EMT.

## Results

### Proteomic studies

**Fig 1:**
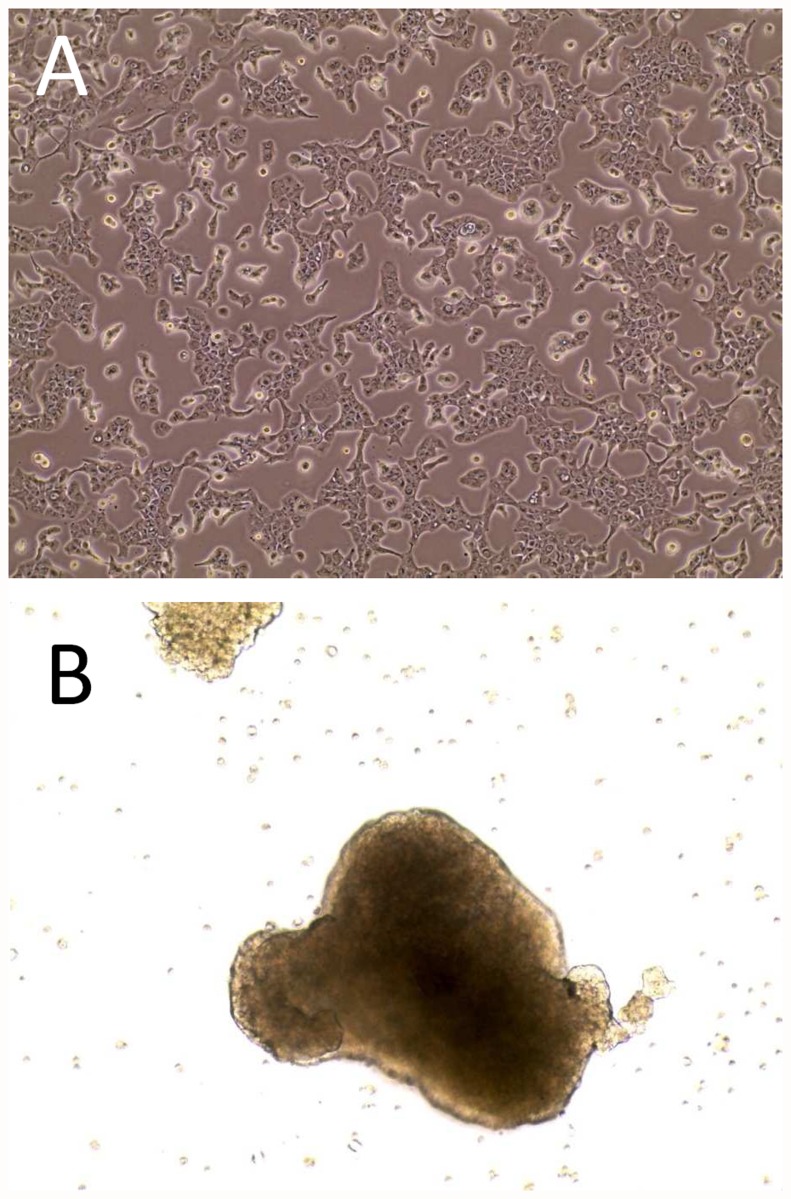
Morphology of cultures of the SK-ChA-1 cell line. Panel A, appearance of the adherent bidimensional culture and, Panel B, appearance of the MCTS (Multicellular Tumor Spheroids). In both instances cells were examined by phase contrast microscopy at 10x magnification.

The morphology of SK-ChA1 cells grown as monolayers is one of adherent pre-confluent cells of a 2D culture grown in resting flasks (Fig. 1A). As to MCTS’s it is one of cellular aggregates (spheroids) obtained under continuous stirring (Fig. 1B). From the same figure it is clearly evident that cellular shapes are also deeply modified, mainly presenting as elongated spindle-like or round-cuboidal in the monolayer and MCTS, respectively. Identical results were also obtained with the MZ-ChA1 cell line, as described elsewhere [19].

**Fig 2:**
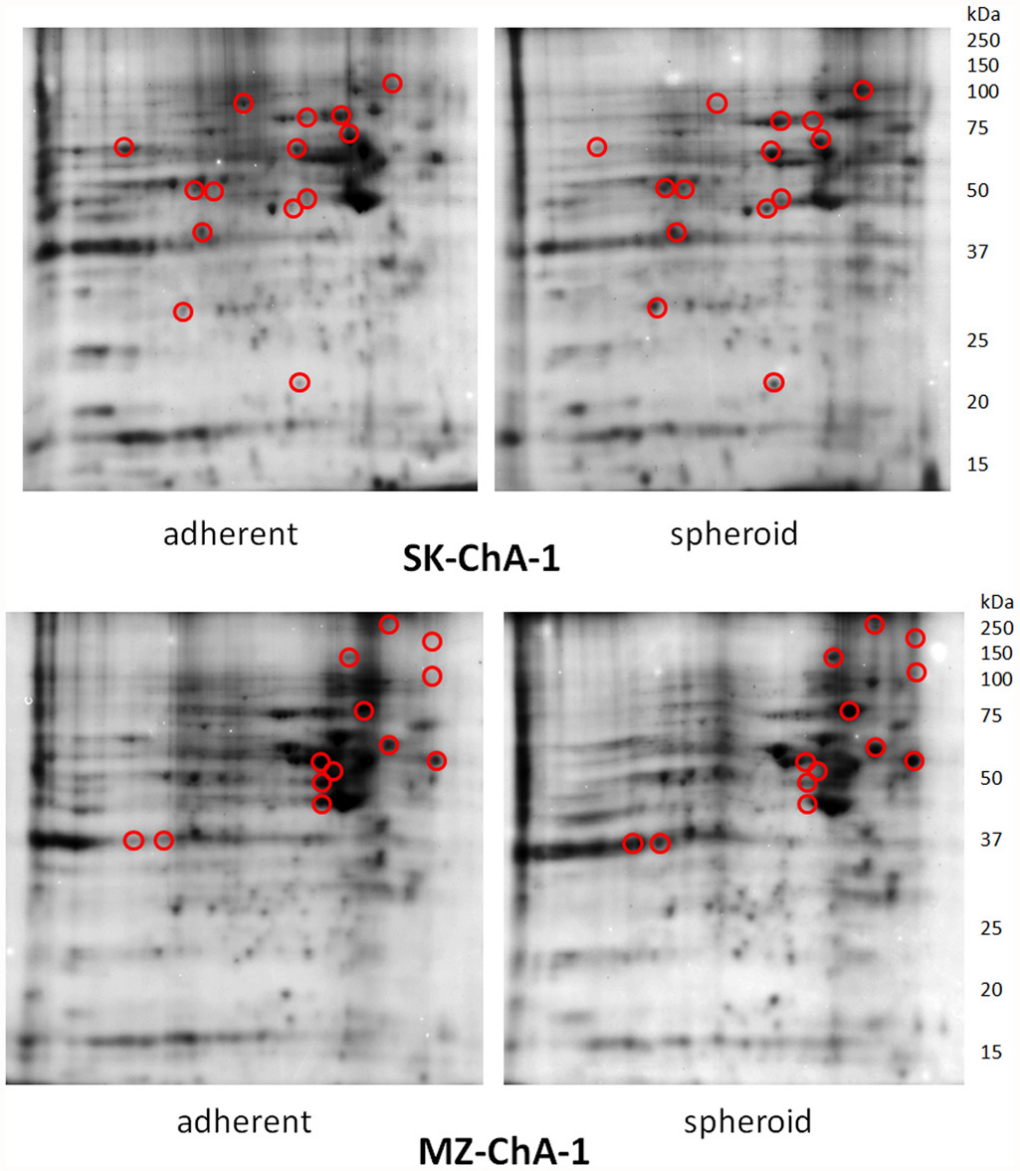
2D-PAGE separation of proteins contained in the cell lysates of SK-ChA-1 and MZ-ChA-1. Proteins were first separated by IEF (pH range 3–10) on a non-linear IEF strip (basic pH at the right) followed by SDS-PAGE in the vertical dimension on a 12.5% gel. Circled protein spots were analyzed by mass spectrometry to compare the relative abundance and results were reported in Table 1.

Homogenates of 2D and 3D cultured cells obtained from both cell lines were submitted to bi-dimensional electrophoresis and yielded different protein patterns. The relevant examples reported in Fig. 2 show appreciable changes in the proteome of confluent cultures (left panel) and in MCTS’s (right panel) of the SK-ChA-1 and MZ-ChA-1 cell lines as finally proved by the analysis of normalized intensity of imaging. Proteins that revealed greater sensitivity to changes are clustered in two pI regions around pH 5 and between pH 7 and 8 (in the pictures the acid pH region is on the right side of each gel). These spots are evidenced by circles on the gel images.

They were selected for identification, by excision from the gels, and for processing via mass spectrometry. We analyzed a total of over 30 proteins in the SK and the MZ systems during the monolayer-to-spheroid conversion. The morphological differentiation was associated with the increased expression of most of the selected proteins, although decreased expression was observed for some of them. These results, which are summarized in Table 1 and include the identity of 22 proteins that we could definitively identify via the proteomic analysis, indicate that some metabolic enzymes, a few cytoskeletal proteins and folding-assisting proteins are affected in cultures grown under different conditions of confluence or in cultures that induce a multicellular spheroid phenotype. We identified enzymes of the glycolytic and Krebs cycle pathways (Triosephosphateisomerase, Glyceraldehydephosphate dehydrogenase, Pyruvate kinase M2 and Isocitrate dehydrogenase); proteins with chaperone (Hsp 70, Hsp 60, calreticulin, ORP 150k), folding (Protein disulfide isomerase and endoplasmin) and antioxidant activity (SOD); inhibitors of proteinases (maspin), organizers of the cytoskeletal activity (Villin, keratin 17, keratin 19, Vimentin) and proteins involved in signaling (phosphatic acid phosphatase, calreticulin and proto-oncogene c-CRK).

### Immunochemical studies of protein expression

**Fig 3:**
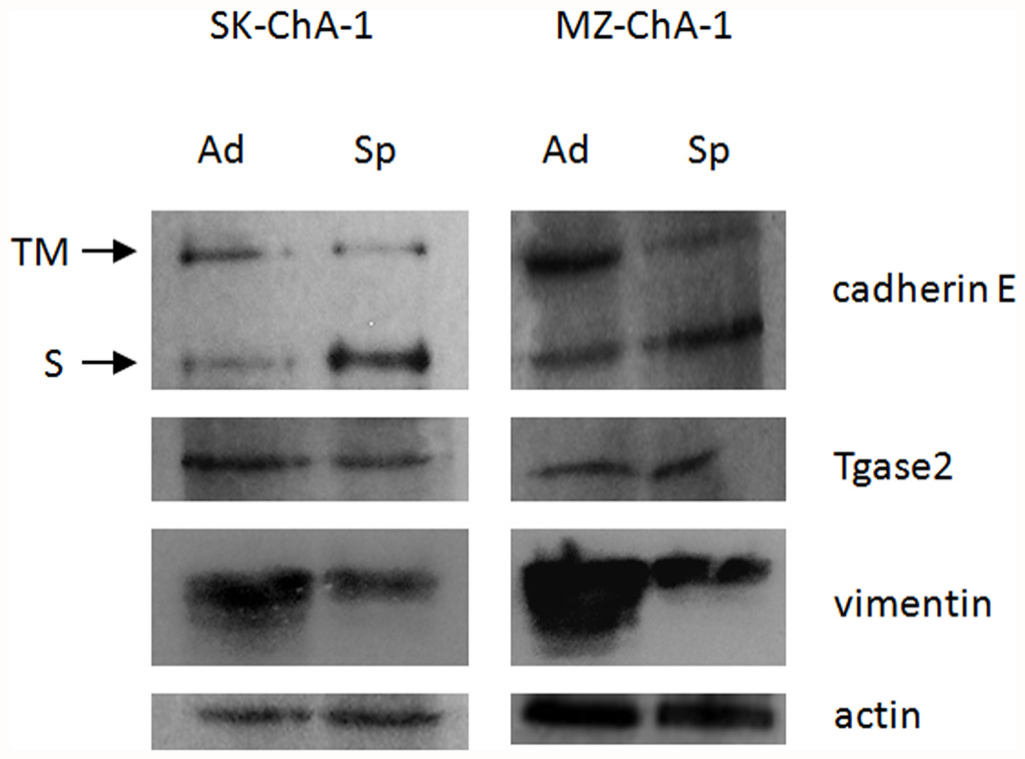
Western blot analysis in adherent and spheroid cells. The figure presents a representative experiment obtained with SK-ChA-1. Ad: adherent; Sp: spheroid; TM: trans-membrane form of cadherin E; S: soluble form of cadherin E.

Based on previously available information, we used Western blots to analyze the expression of a few selected proteins, which have already been correlated with EMT in other types of tumors but were missed in our initial proteomic investigations. These proteins include cadherin E [3,5], Tgase2 and vimentin, whose relation to EMT has been documented in female cancers under endocrine control (ovary, breast and cervix) [6]. In Fig. 3, we have reported the semiquantitive analysis of the expression of cadherin E, Tgase2 and vimentin in immunoblots of lysates from 2D and 3D morphologically differentiated SK-ChA-1 and MZ-ChA-1 cells, which were normalized for protein loading via the amount of immunoreactive actin.

The cadherin E abundance data indicate an imbalance in protein expression between cells grown as a monolayer and those grown as MCTS’s with declining amounts of high molecular weight E-cadherin in cells grown as MCTS, presumably the transmembrane form. Notably, this phenomenon is accompanied by a consistent increase in a peptide that corresponds to a low molecular weight E-cadherin, which usually arises through cadherin solubilization due to proteolysis of the membrane-bound form. Identical results were obtained with the MZ-ChA-1 cell line. These results are consistent with the E-cadherin shift taking place in several tumors during EMT [31] including extrahepatic CC [16]. Vimentin expression was decreased in spheroid cells with respect to adherent cells, confirming the data previously obtained from the 2D PAGE analysis.

### Enzymatic analysis

We have implemented the above immunologic studies by investigating activity of enzymes involved in the carbohydrate metabolism analyzing homogenates of 3 adherent and 3 spheroidal cultures of both MZ-ChA1 and SK-ChA1 for the specific activity of glycolytic and oxidative metabolism. In additional 3 samples we analyzed the expression and activity of Tgase2. We measured in the clarified homogenates the total activity of pyruvate kinase, aldolase, malate dehydrogenase and isocitrate dehydrogenase (ICDH) as notable enzymes of the carbohydrate metabolism. We detected appreciable activity for three enzymes (pyruvate kinase, aldolase and malate dehydrogenase) at comparable levels in lysates from both cell lines grown under conditions leading to adherent or MCTS phenotypes. An exception was NADP-dependent ICDH, which displayed slightly higher specific activity (0.095 *vs*. 0.08 U/mg) in MCTS than in adherent cultures. In addition, we also assessed the activity of the classic NAD-dependent ADP-activated mitochondrial isocitrate dehydrogenase, but it was undetectable in these CC cell lines.

Previously, moderate changes in the activity of Tgase2, which is itself inducible in the SK-ChA-1 and MZ-ChA-1 cell lines, were detected during apoptosis [19]. It is also known that Tgase2 is involved in EMT in other tumors, such as in ovarian cancer [6]. On these bases we analyzed Tgase2 in our setting by immuno-blotting without detecting significant changes in the total amount of intact Tgase2 or appearance of alternatively spliced Tgase2 isoforms [32]. In contrast, we recorded slight differences in the enzyme-specific activity of lysates of adherent cells and spheroid cultures. The putrescine incorporation assays performed at saturating concentrations of calcium yielded specific activity values of approximately 50 nmoles/hr/mg cellular protein in the adherent cells, but values that were 30% lower in MCTS for both cell lines. Furthermore, assays with sub-saturating concentrations of free calcium (0.5 mM) in the absence and presence of GTP proved that the enzyme displayed kinetic properties consistent with those of *wild-type* Tgase2 [26]. This excludes the presence of GTP-insensitive forms arising from alternative splicing [32], with specific activity levels in the presence of 0.5 mM calcium and of calcium/GTP which were respectively 60% and 8–10% at saturating levels of calcium.

### Functional characterization of identified proteins and bioinformatics analysis

**Fig 4:**
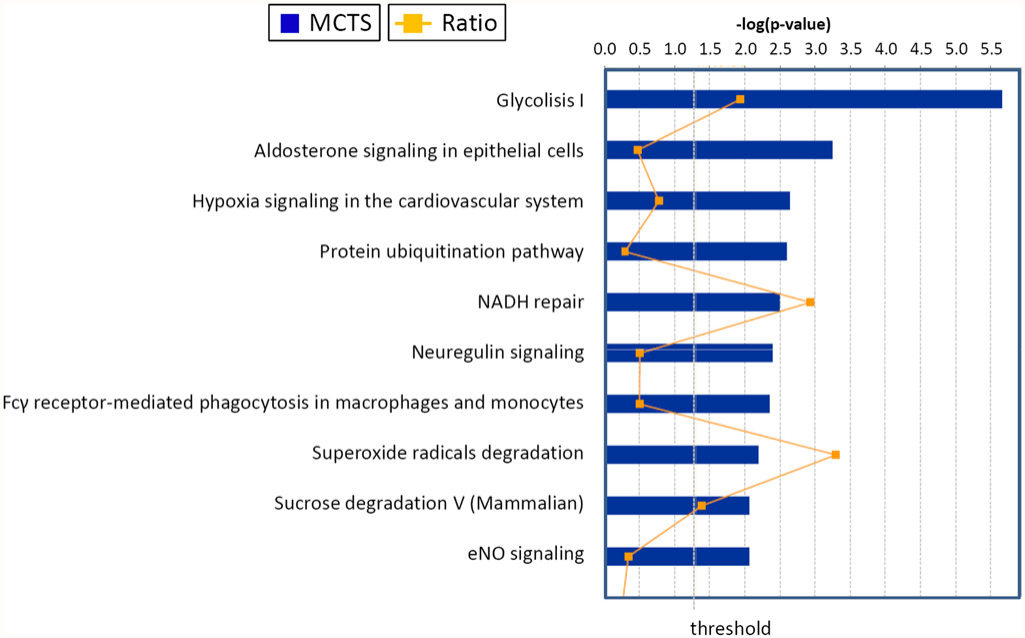
Top canonical pathways identified in cholangiocarcinoma cells EMT. The graph represents host cell pathways with highest score (y-axis) based on the number of differentially regulated proteins using Ingenuity protein analysis. The bar graphs are the pathways most associated with the proteins altered. The orange line graph shows the ratio of the number of molecules from the differentially expressed proteins in EMT that are in the pathway relative to the total number of molecules in the pathway.

We used the proteins we identified as displaying different expressions in response to changes in culture conditions leading to formation of MCTS in CC, to test the biological function using the canonical pathways and network analysis approach by means of the Ingenuity Pathway Analysis (IPA) software [23]. According to the molecular function analysis, most of the proteins were related to some metabolic pathways, protein metabolism, signal transduction, radical degradation, glucose degradation, maintenance of the NAD/NADH oxido-reductive potential (the so-called NADH repair) and other functions. For the proteins taken into account (listed in Table 1), we could identify the related “canonical pathways”, which are depicted in Fig. 4 at statistically significant levels (p ≤ 0.05). Specifically, top values were obtained for glycolysis, aldosterone signaling in epithelial cells, hypoxia signaling in the cardiovascular system, protein ubiquitination, and NADH repair pathways. The additional pathways that were identified at statistically significant levels (p ≤0.05) included neuregulin signaling, Fcγ receptor-mediated phagocytosis in macrophages and monocytes, superoxide radicals degradation, sucrose metabolism in mammals and eNOS signaling (Fig. 4).

**Fig 5:**
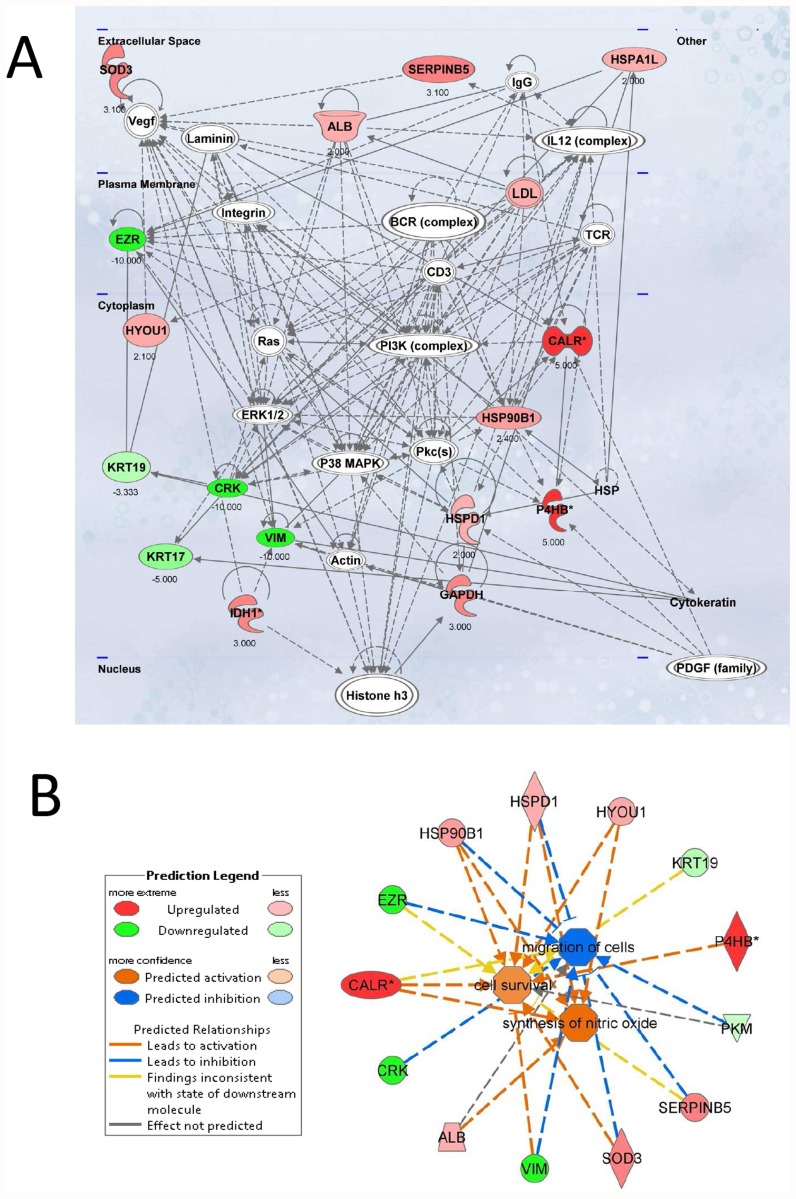
Top network functions affected in the EMT process of cholangiocarcinoma cells. Two relevant networks were generated from EMT-modulated proteins according to the Ingenuity Pathway Knowledge Criteria. A. Cellular compromise, cellular function and maintenance (score = 45). Red, upregulated proteins; green, significantly downregulated proteins; white, proteins known to be in the network but not identified in this study. The color depth indicates the magnitude of the change in protein expression levels. The shape is indicative of the molecular class (i.e protein family). Lines connecting the molecules indicate molecular relationships. Dashed lines indicate indirect interactions, and solid lines indicate direct interactions. The arrow styles indicate specific molecular relationships and the directionality of the interaction. B. Network build up from the three most significant bio-functions (activation Z score >2 or <2): cell survival, synthesis of nitric oxide and migration of cells. The symbols nomenclature is shown in the lower panel.

The scores (-log [p-values]) reflect the probabilities of such associations occurring by chance, with the threshold value for significance set at 1.25. Proteins that changed significantly in spheroids were mapped to two specific functional networks, with each network containing 13 or more “focus” members. The relevant “interactomes” of these two networks were generated from the modulated proteins according to the Ingenuity Pathway Knowledge Criteria and functional associations within those networks (Fig. 5A and 5B). The top networks in which these proteins are involved correspond to (1) cellular damage, function and maintenance and (2) cell survival, synthesis of nitric oxide and migration of cells.

## Discussion

EMT is crucial in metastatic spreading and in the development of therapy resistance in solid tumors, which is likely to occur due to an enrichment in cancer stem cells [8]. EMT occurrence is usually linked to the reprogramming in protein expression via the action of transcription factors (Snail, Slug and Twist) ([33], and references therein) and miRNAs, which together control the shift from E-cadherin to N-cadherin in response to the treatment with cytokines or mechanical stimuli [3,34]. This shift leads to a complete or partial specific EMT “signature”, which dictates biological and clinical outcomes as well as metabolic behavior [29,35].

Additional information about the mechanisms that underlie the EMT process in cancer would be valuable to develop EMT-directed therapeutic strategies in oncology [11, 31, 36]. These treatments would be particularly relevant in the case of tumors that are highly resistant to therapy, such as those of the bile tract, which are characterized by rapid clinical progression and tissue invasion. The studies that have been performed on EMT in CC remain unsatisfactory because these tumors are relatively rare. In addition, CC are also quite heterogeneous, as discussed recently [37], displaying different biological and clinical features depending on the anatomic location (intra-, perihilar or extra-hepatic). Most studies have examined the intrahepatic forms, which might be triggered by specific stimuli, including HCV infection [38] or interaction with stellate cells [39].

In the present study we investigated the pattern of protein expression in two cell lines arising from a differentiated (Mz-ChA-1) and undifferentiated (SK-ChA-1) extra-hepatic cholangiocarcinoma [18] undergoing to MTCS morphology during hypoxic-mechanical stimulation. By immuno-blotting analysis these differential aggregation patterns display changes in the expression of two proteins that are involved in controlling cell proliferation, mobility and invasion, E-cadherin, and mesenchymal differentiation, vimentin. These proteins dictate the biological behavior of CC in patients as finally proved in several reports. For instance, Akira et al. [16] indeed correlated the pattern of cadherin expression with the clinical-pathologic outcome in a number of patients that received surgery for EHCC. Their results suggested that patterns of low/high expression of E-cadherin might represent an important prognostic factor in CC that result in impressive divergences in the Kaplan Maier plots of 5-year survival rates. These plots demonstrated that E-cadherin expression levels are significantly correlated with tumor staging, the occurrence of lymph node metastases and the rate of blood and lymphatic vessel invasion [16]. Notably, increased mortality with 5-year survival rates of 0% was observed for patients whose tumors expressed low levels of E-cadherin, while 5-years survival was 53% for patients whose tumors did express the protein at high levels. In contrast, the expression of N-cadherin did not play any relevant influence on the clinical outcome [16] and it is not even important in MTCS development in at least some cancer cell lines as in LLC1 Lewis lung cancer cells [40], and in A549 lung cancer cells upon exogenous expression of TLE1 [41]. These observations therefore are at variance with the concept that N-cadherin obligatory contributes to the complete signature of EMT, along with an increase of vimentin expression [3].

In our specific case, the results we have obtained show that the assembly of MTCS in the CC cell lines take place with a decline of E-cadherin as well as vimentin. These effects are likely to increase cell growth rate and re-differentiation of cholangiocytes in the 3D aggregation form, which is therefore likely to associate with a more invasive behavior *in vivo*. The decline in E-cadherin expression takes place through the conversion of the high molecular weight form into a smaller one and this process could influence several signaling pathways involved in cell proliferation and morphogenesis. In this perspective, for instance, it has been proposed that the decline in trans-membrane E-cadherin might affect beta-catenin signaling as well as regulate the Twist function in cell-cell recognition, cytoskeleton remodeling and dynamic control of surface adhesion [42, 43]. Notably, the mechanical stimulation we have employed to generate MTCS is also known to affect the function of beta-catenin in other settings [44, 45], thus supporting this possibility. In relation to the decline of vimentin, we must mention that high expression of vimentin has reportedly shown correlation with poor differentiation in cholangiocarcinoma biopsies and EMT phenotype [46] and, conversely, a decline of vimentin would indicate a reversion of the mesenchymal transition.

In order to better characterize the functional features of these cell lines growing in the adherent and in the spheroid state, we employed the high-throughput quantitative proteomics and network analysis using the Ingenuity Pathways Analysis (IPA) to build up the network for a standard biological functions cluster. When comparing protein expression between adherent and 3D growing cultures we observed increasing expression in the spheroids of several enzymes involved in glycolysis, hypoxia signaling in the cardiovascular system, protein ubiquitination pathway, NADH repair, and superoxide radicals degradation. Along with these findings we also noticed down-regulation of 6 proteins, including isoenzymes of pyruvate kinase, vimentin, villin, cytokeratins and the proto-oncogene CRK, which all displayed an expression ratio <0.5 (see Table 1 and Fig. 4). The proteomics results in these two cell lines are not completely superimposing, and this apparent discrepancy might probably relate to the differentiation state of the original tumors from which the cell lines were derived [18] and from the high intrinsic variability of the CC tumors [37]. Our analysis further pointed out changes in aldosterone signaling, which are likely to be significant in the biliary tract because bile production depends on osmotic effects triggered by secretion of both bile acid and sodium [47].

In summary, our results demonstrate that the induction of MCTS by mechanical stirring—the pattern we have selected—is associated with the conversion of E-cadherin and an important decline in vimentin and metabolic reprogramming indicative of an enhanced glycolysis ability, but is not necessarily linked with hypoxia, as experienced in the well-known Warburg effect [48]. As to CC, only a few studies focused on proteomics in this cancer. The information about this cancer is mainly restricted to cadherin expression and reprogramming of keratin synthesis and metalloproteinase activity, as reported in both *in vitro* and *in vivo* studies [49, 50]. Therefore, any comparison with other protein expression data are superfluous, because distinct patterns of protein expression are not necessarily linked to metabolic fluxes (to be investigated by metabolomics, as already reported in some instances in tumor research [51]), although they are a prerequisite. With these limitations, the present data show a reorientation of the metabolic pattern, including changes in the carbohydrate metabolism and presumably changes in the oxygen supply and sensitivity. Further studies are in progress to confirm these hypotheses. Notably, a few of the proteins, for which we documented altered expression, are also known to be affected by mutations in this and other tumors, such as NADP-dependent isocitric dehydrogenase (ICDH). Some of its mutants, which are relatively frequent in brain tumors [52] but also occur in CC [53], affect tumor growth by promoting the accumulation of 2-OH-glutarate. Notably, we used a proteomic approach to prove that the expression of the type 2 isoform increased, with a marginal increase in the specific activity. Conversely, the activity of the classic mitochondrial ICDH isoenzyme (which is NAD-dependent and ADP-activated) was not detectable in the MZ and SK cell lines of this study, as expected in tumors with low rates of aerobic metabolism. Unfortunately, to our best knowledge data on the activity of the mitochondrial aerobic metabolism in the normal biliary epithelium is unavailable to date. Again metabolomic studies might help to understand these metabolic features and address new therapeutic approaches to target angiogenesis.

The insensitivity of Tgase2 to the metabolic perturbations of CC undergoing MCTS formation was unexpected because this enzyme is a well-established player in tumor aggressiveness acting via multiple isoforms of different GTP sensitivity [32]. It is also known to relate to EMT and cell invasiveness [6]. In this context, the GTP binding region has been proposed to be crucial for cell mobility as well as for the sensitivity to antitumoral drug therapy [54]. Further investigations are clearly needed to fully clarify the role of this multifunctional enzyme in tumor biology because it might involve catalytic activities [26] or other signaling pathways [55].

We are confident that our efforts for an improved characterization of the MTCS spheroids form in CC cell lines will prove useful to any future study that aims at developing new therapeutic drugs against these highly aggressive tumors [11, 12].
